# Artificial intelligence readiness and its relationship with thriving at work among Chinese nurses: a latent profile analysis

**DOI:** 10.3389/fpubh.2026.1885808

**Published:** 2026-07-01

**Authors:** Xinyue Chen, Wanyu Ding, Xueyan Wang, Yingying Wang, Shaoyong Ma, Mingfen Tao

**Affiliations:** 1 Graduate School, Wannan Medical University, Wuhu, Anhui, China; 2 Emergency Intensive Care Unit, The First Affiliated Hospital of Wannan Medical University, Wuhu, Anhui, China; 3 School of Nursing, Wannan Medical University, Wuhu, Anhui, China; 4 Department of Nursing, The First Affiliated Hospital of Wannan Medical University, Wuhu, Anhui, China

**Keywords:** artificial intelligence readiness, latent profile analysis, nurses, nursing management, public health, thriving at work

## Abstract

**Objectives:**

To explore the latent profile categories of nurses’ artificial intelligence readiness and analyze the relationship between these categories and thriving at work, so as to provide references for nursing managers to develop targeted management strategies to enhance nurses’ level of thriving at work.

**Methods:**

A cross-sectional survey was conducted from February to April 2026 among 498 nurses from five hospitals in Anhui Province, China. Data were collected using a general information questionnaire, the Medical Artificial Intelligence Readiness Scale, and the Thriving at Work Scale. Latent profile analysis was performed using the 22 artificial intelligence readiness items as manifest indicators. Model selection was based on information criteria, entropy, likelihood-ratio tests, profile size, posterior classification probabilities, parsimony, and interpretability. Chi-square tests, one-way analysis of variance, and multinomial logistic regression were used for exploratory profile comparisons.

**Results:**

Three profiles were identified: low artificial intelligence readiness (*n* = 70, 14.06%), moderate artificial intelligence readiness (*n* = 283, 56.82%), and high artificial intelligence readiness (*n* = 145, 29.12%). Multivariate logistic regression analysis indicated that department, hospital level, frequency of AI use over the past 6 months, and receipt of AI-related training were major influencing factors of the latent categories of nurses’ artificial intelligence readiness (all *p* < 0.05). There were statistically significant differences in the level of thriving at work among nurses in different latent categories of artificial intelligence readiness (*p* < 0.05).

**Conclusion:**

Artificial intelligence readiness among nurses is heterogeneous, and approximately one-third of nurses were classified into a high-readiness profile. The level of thriving at work varies among nurses in different latent categories of artificial intelligence readiness. Nursing managers and public health administrators should consider profile-specific strategies that combine targeted artificial intelligence training, clinical practice support, and career development for nurses.

## 1 Introduction

With the rapid development of science and technology, the emerging new technologies, new paradigms and new formats have provided a strong driving force for the transformation and innovation of various industries. As a pivotal outcome and core driving force in the innovation process, artificial intelligence (AI) technology has been increasingly widely applied in the healthcare field. Meanwhile, nurses as the backbone of the healthcare delivery system, constitute the largest professional group of health care providers and maintain the closest contact with patients (1). A growing body of research has indicated that nurses’ readiness for AI technology is a critical factor influencing the depth of digital and intelligent nursing implementation and the effectiveness of its application (2). Yang et al. (3) found that nurses’ overall AI readiness was at a moderate level, which indicates considerable room for further improvement and underscores the necessity of enhancing their AI readiness. However, the current AI training system for nurses fails to meet the practical demands of clinical work, as training content does not sufficiently integrate the application skills of AI in nursing scenarios. Nursing administrators must develop targeted training programs based on nurses’ varying levels of AI readiness and incorporate these into clinical practice. However, existing studies (4–6) have mainly been conducted from a group-level perspective. These studies have overlooked individual differences and within-group heterogeneity in nurses’ AI readiness, and few attempts have been made to classify its subtypes or perform stratified analysis. Moreover, its association with thriving at work has not been fully substantiated. Consequently, the findings cannot provide robust support for individualized interventions, leading to poorly targeted outcomes and a waste of nursing resources. Given these gaps, this study explores the subtypes of AI readiness and its relationship with thriving at work. Theoretically, this study fills the empirical gap in the research on the relationship between nurses’ digital literacy and positive work states, and enriches the literature in the fields of medical artificial intelligence and occupational positive psychology. Practically, this study provides new perspectives for clinical nursing management and offers solid empirical evidence for designing targeted stratified interventions. Therefore, this study holds significant theoretical and practical value.

## 2 Background

The AI refers to technologies that simulate and extend human intelligence through computer technology, aiming to assist or enhance human capabilities in accomplishing complex tasks (7). Since the concept of AI was first proposed in 1956, its applications in the medical field have encompassed patient care, medical education, and clinical decision-making (8, 9). By leveraging technologies such as deep learning and natural language processing, AI can process and analyze vast amounts of medical data to enhance diagnostic and decision-making accuracy, making it a current research hotspot (10). AI can propel the development of contemporary novel nursing models, primarily used for developing personalized care plans (11–13), real-time monitoring of vital signs and early warning of condition changes (14, 15), enhancing the accuracy and efficiency of nursing documentation (16), and optimizing nurse scheduling and resource allocation (17), thereby improving the quality and efficiency of nursing work. Medical AI Readiness refers to healthcare professionals’ perceived readiness to utilize healthcare AI applications for providing preventive, diagnostic, therapeutic, and rehabilitative services (18). Nurses’ readiness for AI technology plays a critical role in the integration of AI into nursing practice (19, 20).

Thriving at Work refers to a positive psychological state where individuals simultaneously experience vitality and learning in their work (21). Nurses with high levels of thriving at work can reduce occupational burnout (22), decrease negative emotions (23), and enhance job satisfaction (24). Conversely, a lack of thriving at work diminishes work enthusiasm and quality, potentially hindering personal growth and career development (25). Research indicates that when employees actively engage with AI machines, they experience excitement, accomplishment, and a sense of transcendence from mastering new technologies. This can ignite their enthusiasm and vitality for exploring new work domains, thereby enhancing their work engagement (26).

Research on nurses’ AI readiness is growing, yet most studies employ variable-centered approaches that fail to account for heterogeneity across groups. Furthermore, the relationship between different categories of AI readiness and thriving at work remains unclear. While nurses increasingly encounter AI technologies in clinical practice and face rising professional demands, relevant research remains insufficient.

To elucidate the intrinsic relationship between nurses’ AI readiness and thriving at work, this study is grounded in Self-Determination Theory as its core theoretical framework. Self-Determination Theory provides a useful theoretical lens for understanding how AI readiness drives positive work states (27). Originally proposed by Deci and Ryan (28), Self-Determination Theory distinguishes between intrinsic motivation (driven by interest) and extrinsic motivation (driven by external environment or pressure), and posits that the satisfaction of three basic psychological needs for autonomy, competence, and relatedness strengthens individual intrinsic motivation, thereby fostering positive work states. Existing research has confirmed that AI readiness serves as a prerequisite for nurses to effectively utilize intelligent tools and meet their basic psychological needs (29). Furthermore, external environmental factors such as departmental attributes and related training also influence the level of nurses’ AI readiness (3, 30).

In the context of the ongoing digital transformation of nursing practice, artificial intelligence technologies are increasingly being integrated into clinical workflows. For nurses, AI readiness serves as a critical personal resource that enables them to effectively cope with technological changes (31). Specifically, AI tools can take over repetitive tasks, such as documentation and data entry, thereby affording nurses a greater sense of autonomy in their work (32). Meanwhile, nurses with higher levels of AI readiness are more likely to master AI tools proficiently and gain a strong sense of competence at work (4). In addition, AI platforms including electronic health records and teleconsultation systems facilitate interprofessional collaboration, enhance communication and interpersonal connections among healthcare providers, and strengthen their sense of relatedness (33). According to Self-Determination Theory, enhanced autonomy, competence, and relatedness further activate individuals’ intrinsic motivation, manifesting as increased work vitality and a sustained willingness to learn (28), which are the two core dimensions of thriving at work. Conversely, nurses with low AI readiness may struggle to adapt to technological changes, experience frustration, and suffer from reduced autonomy and diminished work vitality. Therefore, AI readiness can be conceptualized as a key personal resource influencing nurses’ thriving at work in the digital healthcare context.

Latent Profile Analysis (LPA) processes data to group individuals with similar characteristics into clusters, thereby identifying population heterogeneity (34). Therefore, this study employs LPA to investigate whether nurses exhibit heterogeneity in their AI readiness. Based on this analysis, we explore the factors influencing latent categories of AI readiness and delve into the relationship between different AI readiness categories and thriving at work. This research aims to provide insights for enhancing nurses’ thriving at work and nursing service quality amid the rapid advancement of AI. Specifically, the study aims to address three questions: (1) What distinct latent profiles of AI readiness exist among nurses? (2) How do nurses characteristics vary across these profiles? (3) Do levels of thriving at work differ among the identified profiles?

## 3 Materials and methods

### 3.1 Study design and participants

This cross-sectional study was conducted from February to April 2026. The reporting of the study was guided by the Strengthening the Reporting of Observational Studies in Epidemiology (STROBE) statement for cross-sectional studies (35).

### 3.2 Setting and participants

Convenience sampling was used in this study. The target population was all registered nurses, including frontline nurses and head nurses, working in clinical departments of five public hospitals in Anhui Province, China, comprising two tertiary hospitals, two secondary hospitals, and one primary hospital. These departments included internal medicine, surgery, obstetrics and gynecology, pediatrics, emergency department, outpatient services, intensive care units, operating rooms, hemodialysis units, and other departments. Following the guidelines stipulated in the “*Tertiary Hospital Accreditation Standards (2025 Edition)*,” the Chinese government classifies hospitals as three levels based on their size, infrastructure, and equipment provisions (36). Specifically, primary hospitals provide preventive, medical, healthcare, and rehabilitation services directly to communities of a certain population, secondary hospitals provide comprehensive medical and health services to multiple communities and undertake certain teaching and research tasks, and tertiary hospitals provide comprehensive medical and health services to several regions and carry out higher education and scientific research missions that extend beyond the regional scope. Nurses have different working environments and practical conditions, ensuring sample representativeness. Inclusion criteria were: (1) hold a license to practice as a nurse; (2) registered nurses with ≥6 months of clinical work experience; (3) informed consent and voluntary participation in this study. Exclusion criteria were: (1) internship, advanced training, rotation nurses; (2) sick leave, vacation, or absentee nurses.

### 3.3 Sample size

For multivariable analyses, the minimum sample size was estimated using the common rule of 5–10 participants per independent variable. Eighteen independent variables were considered (12 demographic and 6 scale dimensions,) yielding a minimum requirement of 180–360 participants; after allowing for a 20% invalid response rate, the minimum target was 225–450 participants (37). Because latent profile analysis generally requires larger samples to obtain stable profile solutions, a sample size above 300 was considered desirable. A total of questionnaires were collected, and valid questionnaires were included in the final analysis, exceeding both requirements.

### 3.4 Measures

#### 3.4.1 General information questionnaires

A researcher-designed questionnaires was used to collect information on the following 12 variables: gender, age, marital status, highest educational attainment, department, professional title, years of experience, hospital level, receipt of AI-related training, interest in AI, frequency of AI use over the past 6 months, types of internet-enabled devices owned.

#### 3.4.2 Medical artificial intelligence readiness scale

Medical AI readiness was assessed using the scale developed by Karaca et al. (18). The scale contains 22 items across four dimensions: cognition (e.g., “I can define the basic concepts of data science”), ability (e.g., “I can harness AI-based information combined with my professional knowledge”), vision (e.g., “I can explain the limitations of AI technology”) and ethics (e.g., “I can use health data in accordance with legal and ethical norms”). Items are scored on a 5-point Likert scale ranging from 1 (strongly disagree) to 5 (strongly agree). The total score ranges from 22 to 110, with higher scores indicating higher medical AI readiness. The original Cronbach’s alpha coefficient was 0.877 and 0.968 in the present study.

#### 3.4.3 Thriving at work scale

Thriving at work was assessed using the corresponding scale developed by Porath et al. (38) and revised into Chinese by Han et al. (39). The scale contains 10 items across two dimensions: vitality (5 items, e.g., “I feel alive and vital”) and learning (5 items, e.g., “I continue to learn more and more as time goes by”). Items are scored on a 7-point Likert scale ranging from 1 (strongly disagree) to 7 (strongly agree), among which item 3 and item 9 are scored reversely. Higher total scores stand for higher levels of work vitality. The Cronbach’s alpha coefficient was 0.860 in the original study and 0.889 in the present study.

### 3.5 Data collection and quality control

In this study, after obtaining administrative approval from participating hospitals, researchers met with the nursing department of each hospital to coordinate the recruitment process and schedule suitable times for data collection without disrupting clinical duties. The Questionnaire Star application (an online data collection platform) was used to develop an anonymous online questionnaire, which was distributed via WeChat (Tencent Holdings Limited). The nursing department then forwarded a unique link or QR code to eligible nurses through departmental WeChat groups. The first page of the questionnaire included a consent form, a brief study introduction, as well as explanations of voluntary participation, confidentiality, and completion instructions. Participants could access the questionnaire only after agreeing to the consent form. The questionnaire was set to require completion of all items before submission, and restricted to one submission per IP address and per device to prevent duplicate responses. A total of 519 questionnaires were returned. Responses with obvious regular response patterns or implausible completion times (<180 s or >720 s, according to the prespecified quality-control rule) were excluded. Finally, 498 valid questionnaires were retained, yielding an effective response rate of 95.95%.

### 3.6 Statistical analysis

Statistical analyses were performed using Mplus 8.3 and SPSS 26.0. Descriptive statistics were used to summarize participant characteristics and scale scores. Continuous variables were presented as mean ± standard deviation (SD), and categorical variables were presented as frequency (n) and percentage (%).

Latent Profile Analysis was conducted in Mplus 8.3 using the 22 AI readiness items as manifest indicators. Models with one to five profiles were fitted sequentially. Model selection was based on the Akaike information criterion (AIC), Bayesian information criterion (BIC), sample-size-adjusted BIC (aBIC), entropy, Lo–Mendell–Rubin adjusted likelihood ratio test (LMR-LRT), bootstrapped likelihood ratio test (BLRT), profile proportions, average posterior probabilities, model parsimony, and substantive interpretability. Lower artificial AIC, BIC, and aBIC values indicate better relative fit; entropy values closer to 1 indicate better classification accuracy; and significant LMR-LRT and BLRT values suggest that the k-profile model fits significantly better than the (*k*-1)-profile model (40–42).

After the optimal profile solution was selected, participants were assigned to their most likely profile based on maximum posterior probabilities. Chi-square tests and one-way analysis of variance (ANOVA) were used for exploratory comparisons across profiles. Variables that were statistically significant in univariate analyses were entered into a multinomial logistic regression model to identify factors associated with profile membership. The low-readiness profile was used as the reference category. A two-sided *p*-value < 0.05 was considered statistically significant.

Because the available dataset was analyzed using maximum posterior probability assignment for subsequent comparisons, the possibility of classification error bias could not be fully eliminated. Therefore, the multinomial regression and ANOVA findings should be interpreted with caution.

### 3.7 Ethical considerations

The study protocol was approved by the Ethics Committee of the First Affiliated Hospital of Wannan Medical University (approval number: (2026) Ethical Review No. 26). All participants provided informed consent before completing the questionnaire. Participation was voluntary, and participants were informed of their right to withdraw at any time without penalty. All data were collected anonymously and used only for research purposes.

## 4 Results

### 4.1 Participant characteristics

A total of 519 questionnaires were distributed, and 498 valid responses were collected, resulting in a response rate of 95.95%. A total of 498 nurses were enrolled, 53 were male and 445 were female. Age: 18–25 years old (*n* = 66), 26–35 years old (*n* = 230), 36–46 years old (*n* = 152), ≥47 years old (*n* = 50). Marital status: single (*n* = 117), married (*n* = 376), divorced (*n* = 4), widowed (*n* = 1). Highest educational attainment: associate degree or below (*n* = 71), bachelor’s degree (*n* = 409), master’s degree and above (*n* = 18). Professional title: nurse (*n* = 74), senior nurse (*n* = 149), supervisor nurse (*n* = 226), associate chief nurse (*n* = 40), chief nurse (*n* = 9). Interest in AI: interested (*n* = 409), uninterested (*n* = 89). Types of internet-enabled devices owned: 1–2 types (*n* = 330), 3 types (*n* = 143), 4 types and above (*n* = 25). More detailed participant characteristics are shown in Table 1.

**Table 1:** Participant characteristics (*n* = 498).

Variable	Category	*n*	Percentage (%)
Gender	Male	53	10.64
	Female	445	89.36
Age (years)	18–25	66	13.25
	26–35	230	46.18
	36–46	152	30.52
	≥47	50	10.04
Marital status	Single	117	23.49
	Married	376	75.50
	Divorced	4	0.80
	Widowed	1	0.20
Highest educational attainment	Associate degree or below	71	14.26
	Bachelor’s degree	409	82.13
	Master’s degree and above	18	3.61
Professional title	Nurse	74	14.86
	Senior nurse	149	29.92
	Supervisor nurse	226	45.38
	Associate chief nurse	40	8.03
	Chief nurse	9	1.81
Department	Internal medicine	89	17.87
	Surgical	96	19.28
	Obstetrics and gynecology	25	5.02
	Pediatric	15	3.01
	Emergency	26	5.22
	Outpatient services	23	4.62
	Intensive care unit	31	6.23
	Operating room	28	5.62
	Hemodialysis Unit	56	11.24
	Other*	109	21.89
Hospital level	Primary hospital	7	1.41
	Secondary hospital	187	37.55
	Tertiary hospital	304	61.04
Years of experience	<1	23	4.62
	1–3	71	14.26
	4–5	28	5.62
	6–10	127	25.50
	11–20	173	34.74
	≥21	76	15.26
Receipt of AI-related training	Yes	133	26.71
	No	365	73.29
Interest in artificial intelligence	Yes	409	82.13
	No	89	17.87
Frequency of AI use over the past 6 month	Almost never	32	6.43
	Occasionally	148	29.72
	At least once a month	113	22.69
	At least once a week	164	32.93
	Daily	41	8.23
Types of internet-enabled devices owned	1–2 types	330	66.27
	3 types	143	28.71
	4 types and above	25	5.02

*Including nurses from the Nursing Department, Rehabilitation Department, and Psychiatry Department.

### 4.2 Scale scores of nurses’ medical AI readiness and thriving at work

The total score of nurses’ medical AI readiness was 68.31 ± 15.95, and the average item score was 3.11 ± 0.73. The total score of nurses’ thriving at work was 49.87 ± 9.01, and the average item score was 4.99 ± 0.90. The results are shown in Table 2.

**Table 2:** Scores of nurses’ AI readiness and thriving at work (*n* = 498).

Item	Total score	Average item score
Medical AI Readiness Scale	68.31 ± 15.95	3.11 ± 0.73
Cognition	22.83 ± 6.73	2.85 ± 0.84
Ability	25.64 ± 6.24	3.20 ± 0.78
Vision	8.96 ± 2.56	2.99 ± 0.85
Ethic	10.89 ± 2.68	3.63 ± 0.89
Thriving at Work Scale	49.87 ± 9.01	4.99 ± 0.90
Vitality	24.42 ± 4.84	4.88 ± 0.97
Learning	25.45 ± 4.70	5.09 ± 0.94

### 4.3 Latent profiles of medical AI readiness among nurses

To determine the optimal model, the 22 item scores of the Medical AI Readiness Scale were taken as manifest indicators to fit one- to five-profile models in sequence. The model fitting results are presented in Table 3. The results revealed that the artificial AIC, BIC, and aBIC values decreased progressively with an increasing number of profiles, with a gentle downward trend starting from the three-profile model. The three-profile model had the highest entropy value of 0.980, which was markedly higher than those of the four-profile (0.964) and five-profile (0.968) models, indicating it achieved the highest classification accuracy. The LMR-LRT values for the four- and five-profile models were both non-significant (*p* > 0.05). Moreover, the average posterior probabilities for all profiles ranged from 0.990 to 0.997, all exceeding 0.90, demonstrating that the three-profile model had good discriminatory power and high classification precision. The average posterior probabilities are presented in Table 4. Consequently, the three-profile solution was finally selected as the optimal model.

**Table 3:** Model-fit indices for latent profile models of AI readiness among nurses (*n* = 498).

Model	AIC	BIC	aBIC	Entropy	LMR-LRT P	BLRT P	Class proportions (%)
1-profile	29670.042	29855.308	29715.650				
2-profile	25512.328	25794.438	25581.777	0.974	0.0153	0.0000	0.237/0.763
3-profile	22113.788	22492.742	22207.078	0.980	0.0002	0.0000	0.141/0.568/0.291
4-profile	21298.119	21773.916	21415.249	0.964	0.2049	0.0000	0.137/0.195/0.378/0.291
5-profile	20594.685	21167.326	20735.656	0.968	0.0656	0.0000	0.163/0.129/0.378/0.137/0.195

AIC, Akaike information criterion; BIC, Bayesian information criterion; aBIC, sample-size-adjusted Bayesian information criterion; LMR-LRT, Lo–Mendell–Rubin adjusted likelihood ratio test; BLRT, bootstrapped likelihood ratio test.

**Table 4:** Average posterior probabilities for the three-profile solution (*n* = 498).

Assigned profile	C1	C2	C3
C1: Low AI readiness	0.997	0.003	0.000
C2: Moderate AI readiness	0.002	0.990	0.008
C3: High AI readiness	0.000	0.010	0.990

C1, low AI readiness; C2, moderate AI readiness; C3, high AI readiness.

### 4.4 Designation of latent profiles of medical AI readiness among nurses

**Figure 1:**
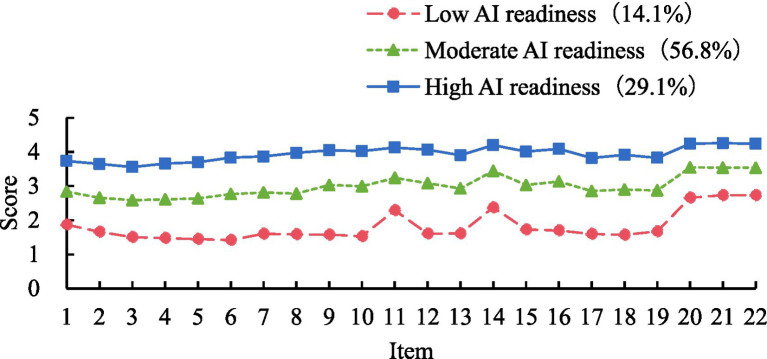
Profile plot of the three latent AI readiness profiles across the 22 manifest indicators.

The three profiles were labeled according to their score levels. C1 represented the low medical AI readiness profile (*n* = 70, 14.06%), with relatively low scores on all items and the lowest overall level, indicating insufficient readiness. C2 represented the moderate medical AI readiness profile (*n* = 283, 56.82%), with moderate scores across all items. C3 represented the high medical AI readiness profile (*n* = 145, 29.12%), which had higher scores on all items and the highest overall level of medical AI readiness. The three profiles showed a consistent low-to-high severity gradient across the total medical AI readiness score and its three dimensions (Table 5). Figure 1 displays the scores for the 22 items across the three potential categories.

**Table 5:** AI readiness dimension scores across the three latent profiles (mean ± SD).

Dimension	Total sample (*n* = 498)	C1 (*n* = 70)	C2 (*n* = 283)	C3 (*n* = 145)	*F*	*P*
Cognition	22.83 ± 6.73	12.54 ± 3.87	21.70 ± 3.60	30.01 ± 4.20	522.69	<0.001
Ability	25.64 ± 6.24	14.40 ± 3.29	24.91 ± 2.52	32.48 ± 2.63	1104.64	<0.001
Vision	8.96 ± 2.56	4.84 ± 1.70	8.63 ± 1.41	11.59 ± 1.39	529.57	<0.001
Ethics	10.89 ± 2.68	8.11 ± 3.06	10.63 ± 2.26	12.73 ± 1.71	103.60	<0.001
Total AI Readiness	68.31 ± 15.95	39.90 ± 7.75	65.86 ± 5.21	86.81 ± 6.90	1431.45	<0.001

### 4.5 Univariate analysis of latent profiles in medical AI readiness among nurses

Table 6 shows that the results of the univariate analysis indicate statistically significant differences (all *p* < 0.05) among the three potential categories of nurses in terms of gender, age, department, hospital level, years of experience, receipt of AI-related training, and frequency of AI use over the past 6 months. Marital status, highest educational attainment, professional title, interest in AI, and types of internet-enabled devices owned were not significantly different across profiles.

**Table 6:** Univariate comparisons of participant characteristics across the three latent profiles (*n* = 498).

Variable	Category	Total (*n* = 498)	C1 (*n* = 70)	C2 (*n* = 283)	C3 (*n* = 145)	*χ* ^2^	*P*
Gender	Male	53 (10.64)	8 (11.43)	22 (7.77)	23 (15.86)	6.648	0.036
	Female	445 (89.36)	62 (88.57)	261 (92.23)	122 (84.14)		
Age (years)	18–25	66 (13.25)	7 (10.00)	32 (11.31)	27 (18.62)	12.884	0.045
	26–35	230 (46.18)	40 (57.14)	129 (45.58)	61 (42.07)		
	36–46	152 (30.52)	13 (18.57)	93 (32.86)	46 (31.72)		
	≥47	50 (10.04)	10 (14.29)	29 (10.25)	11 (7.59)		
Marital status	Single	117 (23.49)	14 (20.00)	62 (21.91)	41 (28.28)	4.593	0.262^△^
	Married	376 (75.50)	55 (78.57)	218 (77.03)	103 (71.03)		
	Divorced	4 (0.80)	1 (1.43)	3 (1.06)	0 (0.00)		
	Widowed	1 (0.20)	0 (0.00)	0 (0.00)	1 (0.69)		
Highest educational attainment	Associate degree or below	71 (14.26)	7 (10.00)	44 (15.55)	20 (13.79)	5.965	0.202
	Bachelor’s degree	409 (82.13)	60 (85.71)	233 (82.33)	116 (80.00)		
	Master’s degree and above	18 (3.61)	3 (4.29)	6 (2.12)	9 (6.21)		
Professional title	Nurse	74 (14.86)	12 (17.14)	34 (12.01)	28 (19.31)	12.565	0.128
	Senior nurse	149 (29.92)	26 (37.14)	80 (28.27)	43 (29.66)		
	Supervisor nurse	226 (45.38)	22 (31.43)	141 (49.82)	63 (43.45)		
	Associate chief nurse	40 (8.03)	9 (12.86)	22 (7.77)	9 (6.21)		
	Chief nurse	9 (1.81)	1 (1.43)	6 (2.12)	2 (1.38)		
Department	Internal medicine	89 (17.87)	6 (8.57)	53 (18.73)	30 (20.69)	32.766	0.018
	Surgical	96 (19.28)	14 (20.00)	57 (20.14)	25 (17.24)		
	Obstetrics and gynecology	25 (5.02)	4 (5.71)	12 (4.24)	9 (6.21)		
	Pediatric	15 (3.01)	4 (5.71)	5 (1.77)	6 (4.14)		
	Emergency	26 (5.22)	6 (8.57)	13 (4.59)	7 (4.83)		
	Outpatient services	23 (4.62)	10 (14.29)	7 (2.47)	6 (4.14)		
	Intensive care unit	31 (6.23)	3 (4.29)	18 (6.36)	10 (6.90)		
	Operating room	28 (5.62)	4 (5.71)	14 (4.95)	10 (6.90)		
	Hemodialysis Unit	56 (11.24)	8 (11.43)	36 (12.72)	12 (8.28)		
	Other*	109 (21.89)	11 (15.71)	68 (24.03)	30 (20.69)		
Hospital level	Primary hospital	7 (1.41)	4 (5.71)	2 (0.71)	1 (0.69)	23.791	0.007^△^
	Secondary hospital	187 (37.55)	19 (27.14)	119 (42.05)	49 (33.79)		
	Tertiary hospital	304 (61.04)	47 (67.14)	162 (57.24)	95 (65.52)		
Years of experience	<1	23 (4.62)	3 (4.29)	10 (3.53)	10 (6.90)	24.848	0.006
	1–3	71 (14.26)	6 (8.57)	40 (14.13)	25 (17.24)		
	4–5	28 (5.62)	7 (10.00)	7 (2.47)	14 (9.66)		
	6–10	127 (25.50)	22 (31.43)	80 (28.27)	25 (17.24)		
	11–20	173 (34.74)	20 (28.57)	99 (34.98)	54 (37.24)		
	≥ 21	76 (15.26)	12 (17.14)	47 (16.61)	17 (11.72)		
Receipt of AI-related training	Yes	133 (26.71)	11 (15.71)	69 (24.38)	53 (36.55)	12.283	0.002
	No	365 (73.29)	59 (84.29)	214 (75.62)	92 (63.45)		
Interest in artificial intelligence	Yes	409 (82.13)	56 (80.00)	229 (80.92)	124 (85.52)	1.633	0.442
	No	89 (17.87)	14 (20.00)	54 (19.08)	21 (14.48)		
Frequency of AI use over the past 6 month	Almost never	32 (6.43)	9 (12.86)	17 (6.01)	6 (4.14)	25.229	0.001
	Occasionally	148 (29.72)	31 (44.29)	77 (27.21)	40 (27.59)		
	At least once a month	113 (22.69)	17 (24.29)	66 (23.32)	30 (20.69)		
	At least once a week	164 (32.93)	12 (17.14)	101 (35.69)	51 (35.17)		
	Daily	41 (8.23)	1 (1.43)	22 (7.77)	18 (12.41)		
Types of internet-enabled devices owned	1–2 types	330 (66.27)	46 (65.71)	191 (67.49)	93 (64.14)	5.872	0.209
	3 types	143 (28.71)	23 (32.86)	80 (28.27)	40 (27.59)		
	4 types and above	25 (5.02)	1 (1.43)	1 (1.43)	12 (8.28)		

*Including nurses from the Nursing Department, Rehabilitation Department, and Psychiatry Department. ^△^ indicates Fisher’s exact test.

### 4.6 Multivariate analysis of latent profiles in medical AI readiness among nurses

Variables with significant differences in univariate analyses were entered into a multinomial logistic regression model. The assignment of independent variables is shown in Table 7. The low medical AI readiness profile (C1) was used as the reference outcome category. The regression results indicated that department, hospital level, receipt of AI-related training, and frequency of AI use over the past 6 months were significantly associated with profile membership (all *p* < 0.05). Detailed results are shown in Table 8.

**Table 7:** Variable assignment method.

Variables	Assignment method
Gender	Male = 1, Female = 2
Age (years)	18–25 = 1, 26–35 = 2, 36–46 = 3, ≥47 = 4
Department	Internal medicine = 1, Surgical = 2, Obstetrics and gynecology = 3, Pediatric = 4, Emergency = 5, Outpatient services = 6, Intensive care unit = 7, Operating room = 8, Hemodialysis Unit = 9, Other = 10
Hospital level	Primary hospital = 1, Secondary hospital = 2, Tertiary hospital = 3
Years of experience	<1 = 1, 1–3 = 2, 4–5 = 3, 6–10 = 4, 11–20 = 5, ≥21 = 6
Receipt of AI-related training	Yes = 1, No = 2
Frequency of AI use over the past 6 months	Almost never = 1, Occasionally = 2, At least once a month = 3, At least once a week = 4, Daily = 5

**Table 8:** Multinomial logistic regression of factors associated with latent profile membership.

Variables	Reference	*β*	SE	Wald *χ*^2^	*P*	OR	95%CI
C2 vs. C1							
Department	Internal medicine						
Pediatric		−1.788	0.887	4.060	0.044	0.167	0.029 ~ 0.952
Operating room		−2.343	0.708	10.937	0.001	0.096	0.024 ~ 0.385
Hospital level	Tertiary hospital						
Primary hospital		−2.851	0.977	8.512	0.004	0.058	0.009 ~ 0.392
Frequency of AI use over the past 6 months	Daily						
Almost never		−2.513	1.214	4.283	0.038	0.081	0.008 ~ 0.875
C3 vs. C1							
Department	Internal medicine						
Emergency		−1.602	0.796	4.049	0.044	0.201	0.042 ~ 0.959
Outpatient services		−2.017	0.755	7.131	0.008	0.133	0.030 ~ 0.585
Hospital level	Tertiary hospital						
Primary hospital		−2.807	1.243	5.097	0.024	0.060	0.005 ~ 0.691
Receipt of AI-related training	No						
Yes		0.850	0.410	4.297	0.038	2.341	1.047 ~ 5.231
Frequency of AI use over the past 6 months	Daily						
Almost never		−2.938	1.265	5.395	0.020	0.053	0.004 ~ 0.632
Occasionally		−2.565	1.146	5.009	0.025	0.077	0.008 ~ 0.727

Only statistically significant predictors from the final model are displayed.

### 4.7 Impact of different latent medical AI readiness profiles on thriving at work among nurses

Thriving at work differed significantly across the three medical AI readiness profiles. Total thriving at work and both dimensions showed an increasing gradient from C1 to C3 (all *p* < 0.001). *Post hoc* comparisons indicated that all pairwise differences were statistically significant, with C1 showing the lowest thriving at work and C3 the highest (Table 9).

**Table 9:** Thriving at work scores across the three latent profiles (mean ± SD).

Profile	Number of cases	Total score	Vitality	Learning
C1: Low AI readiness	70	43.86 ± 0.98	21.70 ± 0.55	22.16 ± 0.52
C2: Moderate AI readiness	283	48.49 ± 0.51^a^	23.66 ± 0.27^a^	24.83 ± 0.26^a^
C3: High AI readiness	145	55.46 ± 0.61^ab^	27.20 ± 0.34^ab^	28.26 ± 0.32^ab^
*F*		57.51	45.37	55.69
*P*		<0.001	<0.001	<0.001

Compared with the low AI readiness group, ^a^*P* < 0.05; Compared with the moderate AI readiness group, ^b^*P* < 0.05.

## 5 Discussion

### 5.1 Heterogeneity of medical AI readiness among nurses

The average item score of nurses’ medical AI readiness was 3.11 ± 0.73, indicating a moderate level, consistent with Eminoğlu et al. (43), suggesting considerable room for improvement. This study identified three latent profiles of AI readiness among clinical nurses: low, moderate, and high AI readiness. More than half of the nurses belonged to the moderate-readiness profile, and nearly one-third belonged to the high-readiness profile. The three profiles represented a level gradient rather than qualitatively distinct response patterns. This finding suggests that AI readiness among nurses may be best understood as a hierarchical continuum in this sample. Such a continuum is meaningful for practice because it can help nursing managers identify nurses who require different levels of AI training, clinical practice support, and career development interventions.

### 5.2 Factors influencing the latent profiles of medical AI readiness

Department was a key factor influencing latent profile membership of nurses’ medical AI readiness. Compared with nurses in internal medicine departments, those in pediatrics, emergency, and outpatient departments were more likely to be classified into the low medical AI readiness profile. This difference may be attributed to the nature of work in different departments (44). According to Self-Determination Theory, the department, as an important external contextual factor, influences nurses’ sense of technological competence and their willingness to engage with technology through differences in job content. Nursing work in internal medicine department involves condition monitoring and nursing documentation, providing nurses with more opportunities to interact with medical information systems, thereby enhancing their cognitive level and application ability (3, 45). In contrast, pediatric nursing places greater emphasis on humanistic care, emergency departments are characterized by a fast pace and high workload, and outpatient nurses have relatively brief contact time with patients, all of which contribute to lower levels of AI technology application (46–48). However, this result should be interpreted with caution, as the sample size of pediatric nurses in this study was small (*n* = 15, 3.01%). Therefore, nursing managers should attend to the technical training needs of departments with lower medical AI readiness, draw on peer mentoring model (49), and encourage nurses with high medical AI readiness to assist those with low readiness, thereby accelerating the development of intelligent nursing.

Hospital level was also a factor associated with latent profile membership of nurses’ medical AI readiness. Compared with nurses in tertiary hospitals, those in primary hospitals were more likely to belong to the low medical AI readiness profile. Tertiary hospitals typically have more comprehensive digital infrastructure and richer training resources, providing nurses with greater opportunities to access AI technology and more frequent usage (50). From the perspective of Self-Determination Theory, comprehensive hardware infrastructure and abundant learning resources in tertiary hospitals help nurses continuously enhance their professional capabilities and satisfy their basic psychological need for competence. In contrast, primary hospitals have relatively weak resource support and technical conditions, resulting in nurses’ insufficient cognitive level and experience with AI. This makes them more likely to be classified into the low readiness profile. This finding is consistent with previous studies, indicating that organizational-level differences in resource allocation significantly inhibit medical AI readiness (31). Nevertheless, this result should be interpreted with caution, as the sample size of primary hospital nurses in this study was small (*n* = 7, 1.41%). Therefore, increased technical support and training investment should be provided to primary hospitals, and a supportive hospital environment should be established to help nurses in these facilities overcome technical barriers and improve their level of medical AI readiness.

Frequency of AI use over the past 6 months was an important factor associated with latent profile membership of nurses’ medical AI readiness. Compared with nurses who used AI daily, those who used AI almost never or occasionally were more likely to belong to the low medical AI readiness profile. Technology use frequency is an important predictor of AI application ability (51). Based on Constructivist learning theory (52), which emphasizes learners’ active construction of knowledge through interaction with their environment based on existing experience and knowledge, rather than passive reception of information, increased frequency of AI use not only enhances technical application ability but also deepens cognitive understanding of AI technology (53). From the perspective of Self-Determination Theory, regular use of AI tools enables nurses to practice repeatedly, consolidate their skills and foster intrinsic motivation for technical learning. Conversely, infrequent use leads to a lack of hands-on practice and independent operation. It hinders technical proficiency and fails to satisfy the needs for competence and autonomy, which ultimately impedes the improvement of AI readiness (54). Therefore, nursing managers should encourage nurses to actively use AI tools in their daily work and life, which is of great significance for cultivating highly qualified nursing professionals.

Receipt of AI-related training was a positively associated factor with nurses’ medical AI readiness. Compared with nurses who had not received AI-related training, those who had received such training were more likely to belong to the high medical AI readiness profile, indicating that relevant training and policy support have achieved positive outcomes. Sommer et al. (55) found that after a hospital established an AI technical support team to provide nurses with immediate assistance and continuous guidance, the efficiency of nurses’ AI tool application increased by 30%. Previous studies have demonstrated that structured systematic training can effectively increase AI knowledge and skills, thereby enhancing nurses’ willingness to use AI (56). Based on Self-Determination Theory, tiered and specialized training helps nurses strengthen connections with colleagues and teams, satisfying their need for relatedness. It also enables them to master AI technologies step by step and fulfill their need for competence. Meanwhile, it improves their autonomy in handling digital work, thereby meeting their need for autonomy. Therefore, nursing managers should establish a stratified AI training system: strengthen basic knowledge training for the low medical AI readiness profile, focus on practical skills training for the moderate readiness profile, and provide innovative application training for the high readiness profile.

### 5.3 The association between AI readiness profiles and thriving at work in nurses

In this study, thriving at work showed a clear positive gradient across the latent profiles of medical AI readiness: nurses in the high-readiness profile had the highest thriving at work scores, whereas those in the low-readiness profile had the lowest. This aligns with the findings of Simsek-Cetinkaya et al. (57), confirming that nurses’ medical AI readiness and thriving at work are closely related. Higher AI readiness may enhance nurses’ learning motivation, increase their vitality and learning behaviors, improve work efficiency by reducing repetitive workload through proficient use of AI tools, and strengthen professional self-confidence and job engagement. Conversely, insufficient AI readiness may diminish nurses’ sense of professional thriving and inhibit their work vitality and emotional engagement.

Therefore, nursing managers should focus on stratified enhancement of nurses’ medical AI readiness by implementing targeted training based on latent profile categories, with particular attention to nurses in the low-readiness profile and specific departments through scenario-based and practice-oriented AI application courses. A supportive hospital environment should be established to optimize AI equipment configuration and usage processes, thereby increasing nurses’ daily exposure to AI technology. In addition, the assessment of medical AI readiness should be integrated into nurses’ professional development system and serve as a reference indicator for evaluating digital transformation effectiveness at the department and hospital levels. Because the present study was cross-sectional, the directionality of this relationship cannot be determined. Longitudinal studies are needed to test whether AI readiness positively predicts nurses’ future thriving at work, whether low thriving at work restricts the improvement of nurses’ AI readiness, or whether both processes occur simultaneously.

### 5.4 Theoretical contributions

This study offers several theoretical contributions.

First, it applies Self-Determination Theory within a unified framework to a sample of Chinese nurses, exploring the relationship between AI readiness and thriving at work. The results are consistent with the idea that AI readiness can function as a key personal resource that satisfies nurses’ basic psychological needs for autonomy, competence, and relatedness, thereby activating intrinsic motivation and promoting thriving at work. Additionally, external environmental factors (such as department type, hospital level, and receipt of AI-related training) contribute to the formation of extrinsic motivation, and the synergy between intrinsic and extrinsic motivation further enhances the positive effect of AI readiness on thriving at work.

Second, the observed latent profile patterns suggest that nurses’ AI readiness exists in three distinct profiles—low, moderate, and high—and that these profiles are progressively associated with levels of thriving at work. This supports a more nuanced understanding of the relationship between AI readiness and thriving at work than a simple linear model, indicating that the association between the two variables is not merely linear but exhibits a clear graded pattern.

Third, this study provides context-specific evidence from five hospitals of different levels in Anhui Province, China. Although the findings should not be generalized to all nurses in China, they provide useful empirical evidence for the important and previously understudied organizational setting of medical AI application scenarios, and offer a theoretical basis for nursing managers to develop stratified intervention strategies.

### 5.5 Implications for nursing education and public health practice

The findings have several practical implications. First, nursing managers should establish a stratified assessment mechanism for nurses’ medical AI readiness, with particular attention to nurses with low and moderate AI readiness. Second, supportive strategies should be tailored to their individual readiness levels: nurses with low readiness may consolidate theoretical knowledge through special lectures and online courses, improve operational proficiency via basic practical training, and gradually build confidence in technology application; nurses with moderate readiness should receive scenario-based training to strengthen their technical competency and receive targeted comprehensive interventions. For nurses with high readiness, advanced training should be provided to enable them to play an exemplary and leading role. Third, the cultivation of thriving at work should be embedded throughout nurses’ career development. Strategies may include clinical AI role-model mentoring, reflective digital practice, career pathway planning, and structured exposure to emerging intelligent nursing scenarios. From a nursing management perspective, improving nurses’ medical AI readiness and enhancing their thriving at work may contribute to a more adaptable, stable, and digitally competent clinical nursing workforce, supporting the high-quality advancement of intelligent healthcare services.

### 5.6 Strengths and limitations

This study has several strengths. First, it recruited a relatively large sample of nurses from five hospitals and adopted a person-centered approach to explore the heterogeneity of medical AI readiness among clinical nurses. Moreover, it further compared differences in thriving at work across distinct latent profiles, which can provide empirical evidence for conducting precise and targeted nursing interventions.

Several limitations should be acknowledged. First, the cross-sectional design precludes causal inference. Second, convenience sampling from hospitals in a single Chinese province limits generalizability. Third, nurses were clustered within different hospitals, but the cluster effect at the hospital level was not modeled. Fourth, all data were collected using self-report questionnaires, which may introduce common-method and social-desirability bias. Fifth, some potentially important confounders, such as anxiety, depression, professional identity, and clinical work pressure, were not measured. Sixth, although the three-profile model showed acceptable classification quality, subsequent analyses used maximum posterior probability assignment and did not fully account for classification error. Future studies should apply three-step methods such as R3STEP and BCH, and should conduct longitudinal, multicenter studies to verify the stability and predictive validity of the identified profiles.

## 6 Conclusion

Based on Self-Determination Theory, this study used latent profile analysis and identified three latent profiles of nurses’ medical AI readiness: low, moderate, and high readiness. The profiles mainly reflected a clear severity gradient, with nurses in higher AI readiness profiles having progressively higher scores on the Thriving at Work scale. Department, hospital level, receipt of AI-related training, and frequency of AI use over the past 6 months were associated with profile membership of nurses’ medical AI readiness. By applying a person-centered approach, this study offers an empirical classification of AI readiness subtypes, enriching prior research that overlooked within-group heterogeneity. Furthermore, this study provides preliminary evidence for a positive association between nurses’ AI readiness and thriving at work, offering an empirical foundation for future research at the intersection of medical AI and occupational positive psychology. Nursing managers should accurately identify nurses at different levels of AI readiness and implement tiered interventions that integrate technical training, environmental optimization, skill guidance, and professional empowerment.

## Data Availability

The raw data supporting the conclusions of this article will be made available by the authors, without undue reservation.
